# Evaluating the Launch of CCSMH's Clinical Practice Guidelines for BPSD

**DOI:** 10.1002/alz70858_104577

**Published:** 2025-12-26

**Authors:** Stacey E Hatch, Jennifer Watt, Zahra Goodarzi, David Conn, Claire Checkland, Dallas Seitz

**Affiliations:** ^1^ CIHR Institute of Aging, Ottawa, ON, Canada; ^2^ University of Toronto, Toronto, ON, Canada; ^3^ Unity Health Toronto, Toronto, ON, Canada; ^4^ Hotchkiss Brain Institute, University of Calgary, Calgary, AB, Canada; ^5^ Baycrest, Toronto, ON, Canada; ^6^ Canadian Coalition for Seniors Mental Health, Markham, ON, Canada; ^7^ Centre for Addictions and Mental Health (CAMH), Toronto, ON, Canada

## Abstract

**Background:**

In March 2024, the Canadian Coalition for Seniors Mental Health launched the Clinical Practice Guidelines (CPG) on the Assessment and Management of BPSD. The goal of our dissemination strategy over the subsequent nine months was to raise awareness of the CPG and enhance healthcare providers' understanding and application of its good practice principles and key recommendations. The primary objective of this poster is to present and evaluate our dissemination strategies.

**Methods:**

Our knowledge translation approach utilized three strategies in English and French. Diffusion activities required minimal customization and included social media posts on LinkedIn, X, Facebook, announcements on CCSMH website, and press releases. Dissemination activities were tailored to audiences including seven scientific conferences, several webinars, and open‐access publications. Application strategies were tailored to participants, such as academic education and training sessions. Evaluation measured cumulative reach of combined activities and synthesized participant feedback.

**Results:**

Diffusion efforts reached approximately 29,481 individuals. The CCSMH website recorded nearly 12,000 visitors, with over 7,800 downloads of the CPG in English and French. Dissemination activities introduced the CPG through English and French webinars, which accumulated over 11,200 views, seven conference presentations attended by more than 900 participants, and open‐access publications. Additionally, over 800 printed copies were distributed. Application strategies included seven academic education and training initiatives, including the 10‐week Project ECHO (Extension of Community Health Outcomes) Care series with over 500 participants, grand rounds at Canadian hospitals and specialty clinics, academic detailing using case studies at conferences, a specialized virtual geriatrics course, and ongoing correspondence with end users. Overall, these knowledge translation activities reached approximately 42,200 individuals. Participant feedback will be presented as graphs.

**Conclusions:**

Feedback highlighted the need for scalable implementation strategies, such as developing clinical summaries in electronic and print formats in both English and French. Our findings emphasize the value of tailored dissemination strategies that engage healthcare providers where they seek evidence‐based information (relevant websites and social media platforms), gather (scientific conferences), and learn (education series and grand rounds). Effective dissemination of the CPG was crucial for the rapid translation of research evidence into actionable recommendations in the assessment and management of BPSD.

